# Blood Pressure Measurement Based on the Camera and Inertial Measurement Unit of a Smartphone: Instrument Validation Study

**DOI:** 10.2196/44147

**Published:** 2023-09-08

**Authors:** Yong-Hoon Yoon, Jongin Kim, Kwang Jin Lee, Dongrae Cho, Jin Kyung Oh, Minsu Kim, Jae-Hyung Roh, Hyun Woong Park, Jae-Hwan Lee

**Affiliations:** 1 Chungnam National University Sejong Hospital, Sejong-Si, Republic of Korea; 2 Deepmedi Research Institute of Technology, Seoul, Republic of Korea

**Keywords:** blood pressure, camera, cuffless, inertial measurement unit, smartphone software, cardiovascular, hypertension, smartphone, measure, accuracy, accurate, software, mHealth, mobile health

## Abstract

**Background:**

Even though several mobile apps that can measure blood pressure have been developed, the data about the accuracy of these apps are limited.

**Objective:**

We assessed the accuracy of AlwaysBP (test) in blood pressure measurement compared with the standard, cuff-based, manual method of brachial blood pressure measurement (reference).

**Methods:**

AlwaysBP is a smartphone software that estimates systolic blood pressure (SBP) and diastolic blood pressure (DBP) based on pulse transit time (PTT). PTT was calculated with a finger photoplethysmogram and seismocardiogram using, respectively, the camera and inertial measurement unit sensor of a commercially available smartphone. After calculating PTT, SBP and DBP were estimated via the Bramwell-Hill and Moens-Korteweg equations. A calibration process was carried out 3 times for each participant to determine the input parameters of the equations. This study was conducted from March to August 2021 at Chungnam National University Sejong Hospital with 87 participants aged between 19 and 70 years who met specific conditions. The primary analysis aimed to evaluate the accuracy of the test method compared with the reference method for the entire study population. The secondary analysis was performed to confirm the stability of the test method for up to 4 weeks in 15 participants. At enrollment, gender, arm circumference, and blood pressure distribution were considered according to current guidelines.

**Results:**

Among the 87 study participants, 45 (52%) individuals were male, and the average age was 35.6 (SD 10.4) years. Hypertension was diagnosed in 14 (16%) participants before this study. The mean test and reference SBPs were 120.0 (SD 18.8) and 118.7 (SD 20.2) mm Hg, respectively (difference: mean 1.2, SD 7.1 mm Hg). The absolute differences between the test and reference SBPs were <5, <10, and <15 mm Hg in 57.5% (150/261), 84.3% (220/261 ), and 94.6% (247/261) of measurements. The mean test and reference DBPs were 80.1 (SD 12.6) and 81.1 (SD 14.4) mm Hg, respectively (difference: mean −1.0, SD 6.0 mm Hg). The absolute differences between the test and reference DBPs were <5, <10, and <15 mm Hg in 75.5% (197/261), 93.9% (245/261), and 97.3% (254/261) of measurements, respectively. The secondary analysis showed that after 4 weeks, the differences between SBP and DBP were 0.1 (SD 8.8) and −2.4 (SD 7.6) mm Hg, respectively.

**Conclusions:**

AlwaysBP exhibited acceptable accuracy in SBP and DBP measurement compared with the standard measurement method, according to the Association for the Advancement of Medical Instrumentation/European Society of Hypertension/International Organization for Standardization protocol criteria. However, further validation studies with a specific validation protocol designed for cuffless blood pressure measuring devices are required to assess clinical accuracy. This technology can be easily applied in everyday life and may improve the general population’s awareness of hypertension, thus helping to control it.

## Introduction

Hypertension is an established major risk factor for various cardiovascular diseases and mortality [1]. Significant efforts have been made to understand the epidemiology, pathophysiology, and associated risks of hypertension to reduce premature cardiovascular morbidity and mortality [2,3]. Thus, the proportion of patients with well-controlled hypertension has increased over the past few decades [4]. High blood pressure can be easily detected at home or in public health centers and is often effectively treated with medications. However, there is a high rate of unawareness of hypertension for a large portion of the total population with hypertension, and the blood pressure control rate of patients with hypertension, especially in low-income countries, remains unsatisfactory. For these people, the early detection and proper management of hypertension are important to prevent future cardiovascular disease, which depends on the accuracy and accessibility of blood pressure measurement methods.

To improve the accessibility of blood pressure measurement, many new devices are being developed for this purpose that are comparable to the conventional, cuff-based, manual method or oscillometric blood pressure measurement devices. Cuffless devices include wristbands, rings, patch systems, and smartphone software; these devices have exhibited acceptable accuracy when compared with conventional blood pressure measurements [5-9]. The principle of cuffless blood pressure measurement is mainly based on the analysis of the pulse transit time (PTT) or photoplethysmography waveform to estimate blood pressure via a linear regression model or machine learning techniques. These methods are easier to use in daily life, and there is no cuff-induced discomfort during their use. However, most new technologies require photoplethysmograms or other sensors to detect the signal waveforms, which may limit their widespread use because of the cost associated with the additional equipment that is required.

The number of smartphone users has increased worldwide, and many people in high-income and low-income countries use them [10]. Most smartphones are equipped with a camera, image sensor, and inertial measurement unit (IMU) sensor; thus, attempts have been made to measure biosignals by using these sensors [11]. If heart movements can be detected by using the IMU sensor of a smartphone and by placing it on an individual’s chest, and if the pulse of the fingertip can be acquired by using the camera of a mobile phone, then the smartphone may be used to estimate the PTT. Furthermore, if blood pressure can be estimated through the PTT obtained by using a smartphone, then blood pressure can be monitored with a smartphone without the use of additional equipment. Therefore, this study assessed a smartphone-based software that estimates blood pressure by using the PTT obtained through the smartphone.

## Methods

### Study Design and Participants

This study was conducted at Chungnam National University Sejong Hospital from March 2021 to August 2021. The enrollment targeted adults aged between 19 and 70 years. Those who met the following conditions were excluded: (1) individuals who were unable to use the test or reference blood pressure measurement device for up to 30 minutes in a sitting position (2) and individuals who had a history of cardiac arrhythmias or peripheral vascular disease. We determined the number of participants according to a validation guideline, which states that at least 85 patients are required for an Association for the Advancement of Medical Instrumentation/European Society of Hypertension/International Organization for Standardization (AAMI/ESH/ISO) validation study [12]. At enrollment, gender, arm circumference, and blood pressure distribution were also considered according to the AAMI/ESH/ISO guideline [12,13].

**Figure 1.**
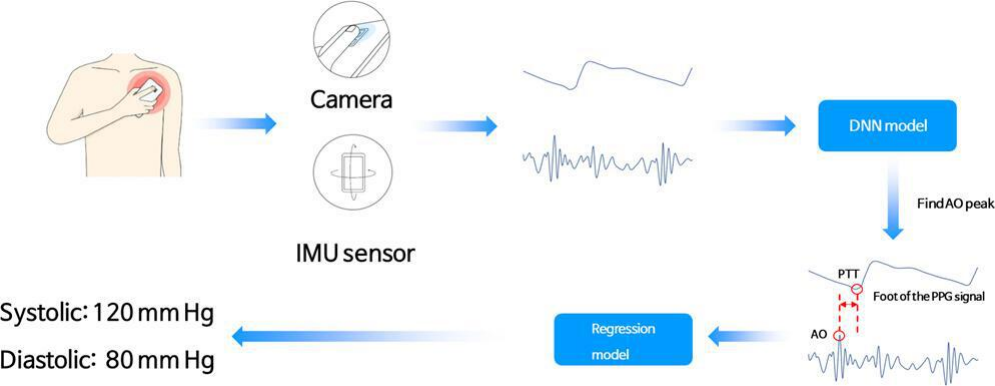
Mechanism and application of the test device. AO: aortic opening; DNN: deep neural network; IMU: inertial measurement unit; PPG: photoplethysmography; PTT: pulse transit time.

The test device, AlwaysBP (Deepmedi Inc), is a smartphone software that estimates a person’s systolic blood pressure (SBP) and diastolic blood pressure (DBP) based on the PTT. The blood pressure estimation model was designed to include the following two steps: (1) calculation of the PTT by using seismocardiography and photoplethysmography via the test device and (2) input of the acquired PTT into the blood pressure estimating equations (Figure 1). PTT was calculated as the time interval between the aortic valve opening on the seismocardiogram, as detected by the IMU sensor, and the onset of the photoplethysmography waveform on the index finger, as detected by the camera of the smartphone. These seismocardiography and photoplethysmography signals were acquired for 20 seconds in each measurement. An infinite impulse response band-pass filter was used for seismocardiography (5-45 Hz) and photoplethysmography (0.8-8 Hz) to denoise the raw signal. The filtered seismocardiography signal was then ensemble-averaged after segmenting the interval between the onset of each wave of the photoplethysmography signal and 450 milliseconds after the onset. This ensemble-averaged signal was input into a deep neural network model to determine the aortic valve opening time accurately. The manufacturer pretrained this deep neural network model, and the same model was used for all participants. After PTT calculation, SBP and DBP were estimated with the equations introduced by Bramwell-Hill and Moens-Korteweg, as follows [14]:


(1) $$SBP=DBP+PP_{0}{\left(\frac{PTT_{0}}{PTT}\right)}^{2}$$

(2) $$DBP=MBP_{0}+\frac{2}{\gamma }\;ln\left(\frac{PTT_{0}}{PTT}\right)-\frac{PP_{0}}{3}{\left(\frac{PTT_{0}}{PTT}\right)}^{2}$$

To determine the input parameters of the equations, a calibration process was carried out 3 times for each participant [14]. The PTT was measured by using the test device during calibration, and SBP and DBP were also measured simultaneously by using the cuff-based reference device. Pulse pressure (PP) and mean blood pressure (MBP) were calculated based on the SBP and DBP values. Based on the first and second calibration processes, the average PTT value obtained by the test device was entered as PTT_0_, and the average PP and MBP measured by cuff-based blood pressure measurement device were entered as PP_0_ and MBP_0_, respectively. The PTT value obtained from the third calibration was input as the PTT of the formulas, and SBP and DBP values were input as the SBP and DBP, respectively; moreover, the γ value was updated according to the results of the calculated equations (before the correction, γ was determined to be 0.031 for individuals younger than 40 years and 0.09 for those older than 40 years) [15]. After the correction was completed, when measuring blood pressure for the primary analysis, the averages of the MBP, PP, and PTT values from the three calibration processes were input as MBP_0_, PP_0_, and PTT_0_, respectively, and the γ obtained from the third calibration was used in the equations. This equation model was implemented in the AlwaysBP app and used to measure blood pressure at the initial visit and at follow-up. The test blood pressure measurement software was installed and used in a Galaxy S10 smartphone (Samsung Electronics) throughout this study. The test software has yet to be released on the app market for commercial use.

### Data Acquisition and Blood Pressure Measurements

The basic demographics, including past medical history, comorbidities, and drug use, of the participants were assessed through an interview with the physician and recorded on a dedicated electronic case reporting form. The participants sat in a comfortable position for at least 5 minutes before measuring blood pressure. During the blood pressure measurement, the participant’s back and arms were supported, their left arm was placed at heart level, their legs were not crossed, and their feet were placed flat on the floor. Dialogue and other interferences were prohibited throughout the measurement. Calibration of the test device was performed just before the measurement of the blood pressure.

The test blood pressure was defined as the blood pressure value measured by the test device (AlwaysBP), and the reference blood pressure was defined as the blood pressure value measured by the reference blood pressure device (InBody). The reference SBP and DBP were measured, using the participant’s left arm, by 2 trained nurses who were blind to each other’s readings. A double-headed stethoscope (Y-tube) was used for the simultaneous auscultation of the brachial pulse, and the blood pressures at the first and fifth Korotkoff sound were recorded as SBP and DBP, respectively. The average blood pressure values measured by the two observers were considered the reference blood pressures for the analysis. The test blood pressure was measured simultaneously with the reference blood pressure. The participant’s right hand held the test device, and the index finger was positioned to cover the camera lens on the back of the smartphone to obtain the finger photoplethysmography signal. Concomitantly, the seismocardiography signal was obtained by placing the front of the smartphone on the participant’s anterior chest wall. This position was maintained for 20 seconds, and if the participant’s position was inadequate for proceeding with the study or blood pressure measurement, the research personnel advised the participant to return to the correct position. If an error occurred because of the poor signal quality of the test blood pressure device resulting from the insufficient color change of the finger to red (a result of the LED light being below a predetermined threshold), blood pressure measurement was attempted again until the error did not occur.

### Study Protocol

**Figure 2.**
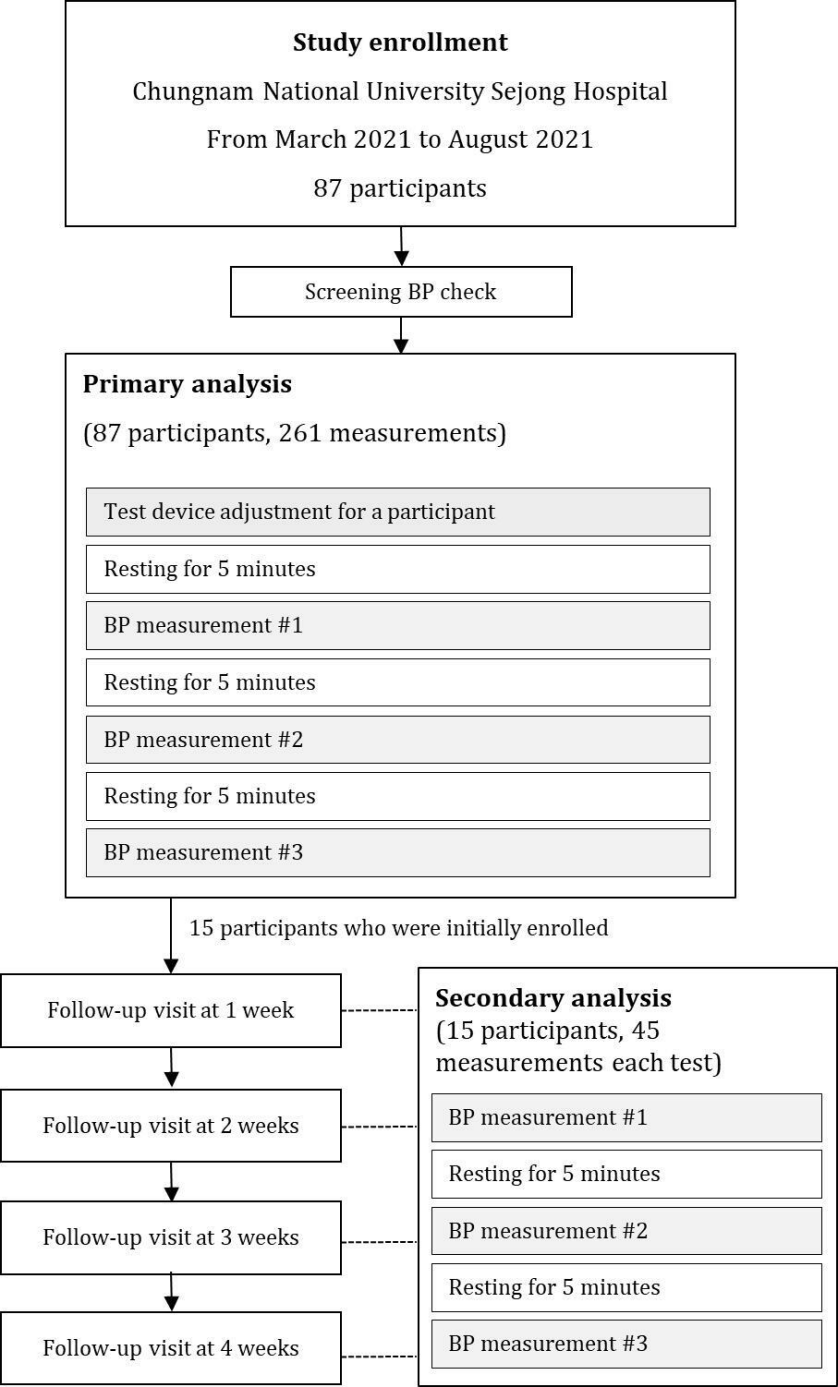
Study flowchart. BP: blood pressure.

This study consisted of 2 parts (Figure 2). The primary analysis aimed to evaluate the accuracy of the test device compared with the reference device for the entire study population (N=87). In turn, the secondary analysis was performed to confirm the stability of the test device for up to 4 weeks after the initial calibration and blood pressure measurement.

For the primary analysis, blood pressure was measured 3 times for each participant after individualized calibration. Calibration was performed by entering the measured reference blood pressure value into the test device after applying the test and the reference devices at the same time. This process was performed 3 times consecutively, without a break. Afterward, 5 minutes of rest were allowed after completion of the calibration. Subsequently, blood pressure was measured 3 times per participant, with an interval of 5 minutes for resting. As a result, a total of 261 pairs of test-reference blood pressure sets from 87 participants were obtained for SBP and DBP, respectively.

Blood pressure was measured and recorded weekly for 4 weeks in 15 participants who were initially enrolled in this study to investigate the stability of the test device over time. Blood pressure measurements performed during the follow-up were carried out identically to the initial blood pressure measurements, with the exception that no calibration was performed.

### Statistical Analysis

Baseline characteristics were expressed as percentages for categorical variables and as means with SDs for continuous variables. Differences between test and reference blood pressure values in the primary and secondary analyses were expressed as means and SDs. To evaluate the correlation between the test and reference blood pressures, the *P* value and Pearson correlation coefficient (*r*) were calculated. Bland-Altman plots were generated to compare the distribution of differences in SBP and DBP between the test and reference devices. All reported *P* values were 2-sided, and significance was set at *P*<.05. All statistical analyses were performed by using R software (version 4.1.1.; R Foundation for Statistical Computing).

### Ethics Approval

All participants participated voluntarily in this study and provided written informed consent. The study protocol was approved by the Institutional Review Committee of Chungnam National University Sejong Hospital (approval number: 2020-10-023). This study was conducted in accordance with the relevant guidelines and regulations.

## Results

### Baseline Characteristics

Among the 87 study participants, 45 (52%) individuals were male, and the average age was 35.6 (SD 10.4) years. The mean height and weight were 168.2 (SD 8.1) cm and 75.2 (SD 17.9) kg, respectively. Further, 14 (16%) participants were diagnosed with hypertension and were taking antihypertensive drugs prior to enrollment. The most used drug to manage hypertension was an angiotensin II receptor blocker. The mean screening SBP and DBP were 134.2 (SD 20.3) mm Hg and 82.8 (SD 15.2) mm Hg, respectively (Table 1).

**Table 1.** Baseline characteristics of the participants (N=87).

Characteristics		Value
Age (years), mean (SD)		35.6 (10.4)
Men, n (%)		45 (52)
Height (cm), mean (SD)		168.2 (8.1)
Weight (kg), mean (SD)		75.2 (17.9)
Arm circumference (cm), mean (SD)		30.0 (3.8)
Hypertension with medications, n (%)		14 (16)
Angiotensin II receptor blockers, n (%)		11 (13)
Beta-blocker, n (%)		3 (3)
Calcium channel blocker, n (%)		9 (10)
Diuretics, n (%)		3 (3)
Diabetes mellitus, n (%)		3 (3)
Dyslipidemia, n (%)		5 (6)
**Blood pressure at screening**		
	SBP^a^ (mm Hg), mean (SD)	134.2 (20.3)
	DBP^b^ (mm Hg), mean (SD)	82.8 (15.2)
	HR^c^ (beats per minute), mean (SD)	81.2 (10.4)
	SBP≥160 mm Hg, n (%)	9 (10)
	DBP≥100 mm Hg, n (%)	14 (16)
	SBP≤110 mm Hg, n (%)	9 (10)
	DBP ≤70 mm Hg, n (%)	17 (20)

a SBP: systolic blood pressure.

b DBP: diastolic blood pressure.

c HR: heart rate.

### Primary Analysis

**Figure 3.**
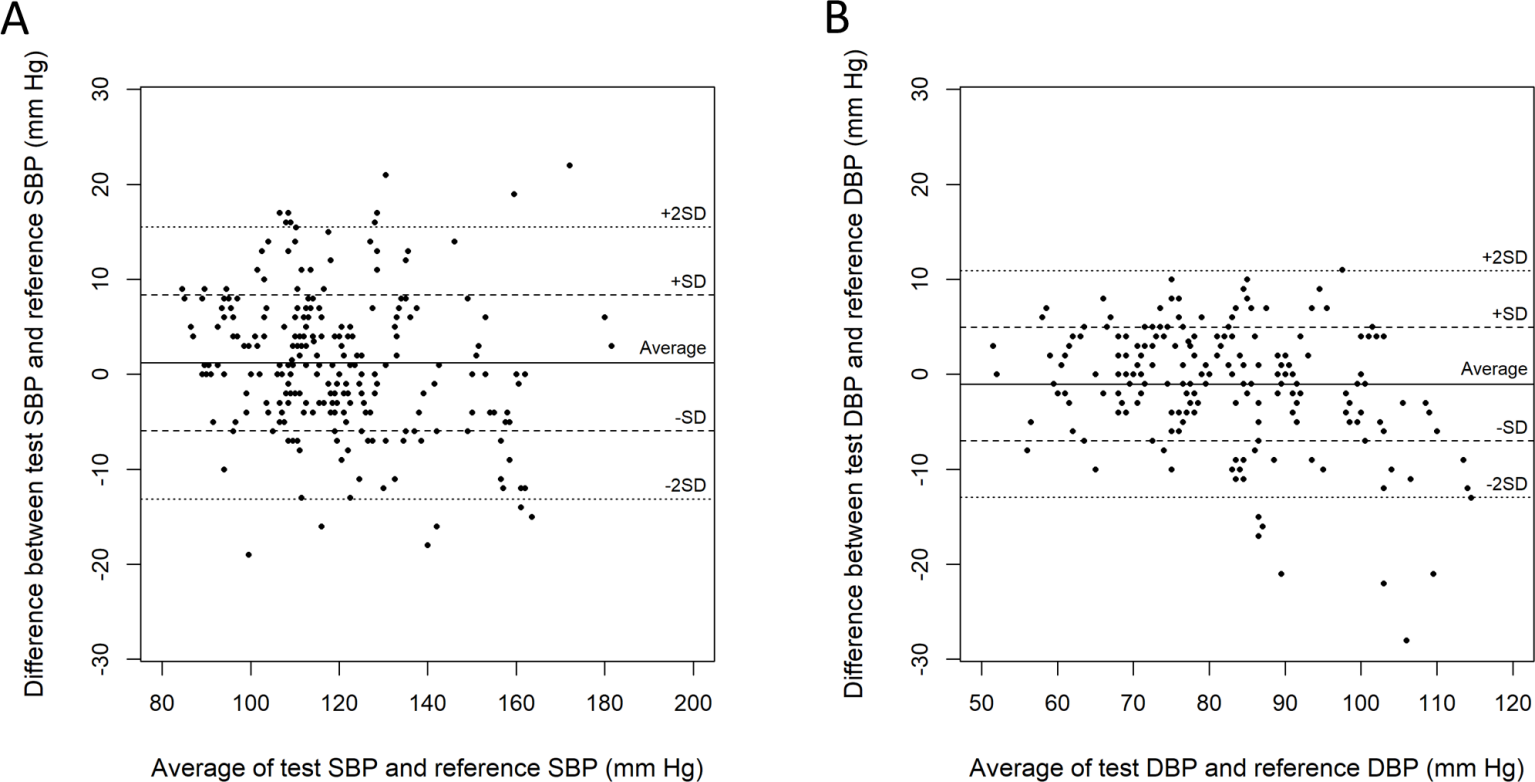
Bland-Altman plots of the SBPs and DBPs measured using the test and reference devices. (A) Bland-Altman plot of the test and reference SBPs. (B) Bland-Altman plot of the test and reference DBPs. DBP: diastolic blood pressure; SBP: systolic blood pressure.

The mean test SBP was 120.0 (SD 18.8) mm Hg, and the mean reference SBP was 118.7 (SD 20.2) mm Hg (Table 2). The difference between the test and reference blood pressures was 1.2 (SD 7.1) mm Hg. The absolute differences between the test and baseline SBPs were <5, <10, and <15 mm Hg in 57.5% (150/261), 84.3% (220/261), and 94.6% (247/261) of the measurements, respectively (Figure 3). In the Bland-Altman plot, of the 261 measurements, 15 (5.7%) were outside 2 SDs of the differences between test and reference SBPs. The DBP values of the test and reference devices were 80.1 (SD 12.6) mm Hg and 81.1 (SD 14.4) mm Hg, respectively. The difference was −1.0 (SD 6.0) mm Hg, but the difference became progressively more pronounced at higher blood pressure levels. The absolute differences between the test and the reference DBPs were <5, <10, and <15 mm Hg in 75.5% (197/261), 93.9% (245/261), and 97.3% (254/261) of the measurements. In the Bland-Altman plot, of the 261 measurements, 10 (3.8%) were outside 2 SDs of the differences, mostly for high blood pressure values.

**Table 2.** Differences between the test and reference blood pressures among 87 participants (261 measurement pairs in the primary analysis).

		SBP^a^, mm Hg	DBP^b^, mm Hg	HR^c^, beats per minute
Test device, mean (SD)		120.0 (18.8)	80.1 (12.6)	75.2 (10.4)
Reference device, mean (SD)		118.7 (20.2)	81.1 (14.4)	75.4 (10.1)
Difference, mean (SD)		1.2 (7.1)	−1.0 (6.0)	−0.2 (6.9)
**Coefficient of variation for each participant, %**				
	Test device	1.3	0.4	4.9
	Reference device	3.1	3.2	4

a SBP: systolic blood pressure.

b DBP: diastolic blood pressure.

c HR: heart rate.

### Secondary Analysis

Table 3 lists the results of the follow-up measurements for the test and reference blood pressure values in 15 participants, which were used in the secondary analysis. The differences at the baseline were 1.7 (SD 6.9) mm Hg (*P*=.10) and −2.0 (SD 5.3) mm Hg (*P*=.01) for SBP and DBP, respectively. After 4 weeks, the differences between SBP and DBP were 0.1 (SD 8.8) mm Hg (*P*=.96) and −2.4 (SD 7.6) mm Hg (*P*=.04), respectively.

**Table 3.** Differences between the test and reference blood pressures among 15 participants during the 4-week follow-up (45 measurement pairs per week in the secondary analysis).

		Baseline	1 week	2 weeks	3 weeks	4 weeks
**SBP^a^**						
	Difference (mm Hg), mean (SD)	1.7 (6.9)	1.1 (6.8)	1.0 (7.6)	1.2 (8.7)	0.1 (8.8)
	*P* value	.10	.31	.36	.37	.96
**DBP^b^**						
	Difference (mm Hg), mean (SD)	−2.0 (5.3)	−1.6 (7.5)	−3.2 (6.8)	0 (8.1)	−2.4 (7.6)
	*P* value	.01	.15	.003	.97	.04

a SBP: systolic blood pressure.

b DBP: diastolic blood pressure.

## Discussion

### Principal Findings

This study assessed the accuracy of a smartphone-based blood pressure measurement software (AlwaysBP) compared with the standard, cuff-based, manual method of brachial blood pressure measurement. Our results revealed that the test device showed acceptable accuracy for both SBP and DBP, and the difference between the test and reference SBP values was stable over the 1-month follow-up. The DBP values of the study device were not stable for 1 month.

The development of various cuffless blood pressure monitors has allowed for the measurement of blood pressure easily and comfortably. Such devices are expected to improve the penetration rate of blood pressure measurements, which is very important for lowering the rates of hypertension unawareness and uncontrolled hypertension after diagnosis. Several smart devices that can measure blood pressure without the use of a cuff have been developed. The most commercially available devices are smartwatch devices, which work mainly by acquiring photoplethysmography signals and estimating blood pressure via a machine learning algorithm based on these signals or PTT calculation [5,7,9,15,16]. The greatest advantage of smart devices is their ability to measure blood pressure continuously without interference; as such, they can be used to monitor blood pressure during rest, work, and exercise. In addition, they facilitate 24-hour ambulatory blood pressure monitoring by analyzing the trend and pattern of blood pressure change over 24 hours. Although controversies remain regarding their accuracy, they represent good alternatives to cuff-based, 24-hour ambulatory blood pressure monitoring if their accuracy is improved. Devices that measure blood pressure by using only a smartphone, without additional devices, have also been developed. With OptiBP (Biospectal), the photoplethysmography signal of the finger is measured by using a smartphone camera, and a pretrained deep learning model is used to estimate blood pressure based on the photoplethysmography signal [6,17]. Similar to the test device used in our study, the OptiBP software does not require an additional device; thus, it has the advantage of significantly improving the accessibility to blood pressure measurement.

In our study, the test device estimated SBP and DBP based on the PTT. The advantage of PTT-based blood pressure measurement over photoplethysmography analysis–based blood pressure estimation has not been elucidated fully. However, there is a huge body of evidence that PTT is associated with blood pressure [18-20]. Moreover, the PTT can be used to assess vascular stiffness, arterial atherosclerosis, and the risk of future cardiovascular events [21]. The software system used in our study does not require additional devices to measure both photoplethysmography signals and cardiac signals for PTT calculation. Moreover, because the AlwaysBP software collects photoplethysmography and seismocardiography signals simultaneously, it may detect an abnormal heart state more accurately than other devices that use photoplethysmography alone [22]. Therefore, this software may play a role in improving the early detection of hypertension and arterial stiffness and in the prediction of cardiovascular events among the general population.

The existing blood pressure measuring devices requiring calibration also have limitations and therefore need to be improved. Changes in blood pressure within an individual are not usually considered in calibration and testing. Thus, there have been debates regarding the problems in validating cuffless blood pressure measuring devices that require calibration, because the time between calibration and testing is insufficient to account for individual differences in blood pressure [23]. Our system also relies on a single individualized calibration, which may not be suitable for longtime use. Calibration was done to calculate γ in the equations, and this value can be affected by age, sex, height, and vascular stiffness. The single-calibration results of the initial measurements were used for a long time. Even though the differences in SBP values between the test and reference devices among 15 participants over 1 month showed satisfactory results, the SD tended to become more prominent at follow-up. Additionally, DBP values were not stable during follow-up in our study population. Our results showed that accuracy may decrease over time, suggesting that a regular calibration process for long-term users may be necessary. This decrease in accuracy may have been due to changes in physiological properties, such as vascular stiffness and atherosclerosis, that vary at different rates in individuals over time. There will also be issues about the necessity of regular calibration and how often those calibrations will be needed for accurate blood pressure measurements. Ultimately, a calibration-free system is needed, but the development of calibration-free devices is still ongoing [24]. To minimize the calibration issues, new devices that can be used without calibration should be developed. Moreover, the test DBPs showed relatively poor correlations at high blood pressures in this study, although the reasons underlying this finding are unclear. PTT calculation via smartphones is different from conventional PTT calculation, which involves electrocardiographic and photoplethysmographic sensors that are used in clinical practice and have higher resolutions. The inherent limitations of using a smartphone-only blood pressure measurement system result in potential accuracy issues, rendering such systems inferior in accuracy when compared to other smart devices, such as smartwatches with electrocardiographic and photoplethysmographic sensors. Further, we used a set of commonly used equations for calculating blood pressure with PTT. This equation set may not have been fit for our data, which were acquired via smartphones. Therefore, additional studies are needed, and an update of the algorithm may be necessary.

Due to the inherent features exhibited by cuffless blood pressure measuring devices, it is imperative to establish more specialized criteria for validating such products [25]. The existing criteria cannot account for the capability of these devices to accurately monitor blood pressure fluctuations in response to positional changes, physical activity, emotional stress, and prolonged periods, which can significantly influence individuals’ blood pressure levels. Consequently, it is essential to subject future devices to rigorous testing with a dedicated validation protocol tailored for cuffless devices. These protocols should emphasize the ability to effectively track blood pressure variations for conditions commonly encountered in daily life.

### Limitations

This study had several limitations. First, it was conducted at a single center in Korea. The device algorithm was based on the Korean population during development, and this study was also conducted with Koreans. Second, although study participants were enrolled according to the AAMI/ESH/ISO protocol, we understand that this protocol should not be used for a cuffless device; therefore, further study is required with a validation protocol specifically designed for cuffless blood pressure devices. Third, both arms were used to detect test and reference blood pressure values simultaneously. Although participants with a history of peripheral vascular disease were not enrolled, the difference in reference blood pressures between both arms was not assessed prior to this study. Fourth, despite meeting the validation criteria suggested by the AAMI/ESH/ISO guidelines, our device’s accuracy may not be sufficient for populations with high DBPs, as shown by the study results. Fifth, our study primarily included young and healthy participants; thus, the accuracy and applicability of our device for older and comorbid populations may be limited. Sixth, our study did not include the average agreement between the two observers and the frequency of device errors; therefore, further study is required to assess the functional consistency and reliability of the AlwaysBP software. Lastly, our data did not include information regarding the skin pigmentation of participants, which can have effects on the signal to noise ratio. Although our study exclusively recruited individuals of East Asian ethnicity and used LED technology from smartphones to obtain high-quality photoplethysmography data from participants’ fingers, the influence of an individual’s skin pigmentation cannot be disregarded. Hence, an evaluation of the accuracy of the study device based on variations in skin pigmentation is necessary. Further investigations involving larger sample sizes with diverse comorbidities and skin colors are also required. Such additional studies should be conducted by an independent group for transparency and to mitigate potential bias [26].

### Conclusion

This study assessed the accuracy of the AlwaysBP smartphone software, which was used to estimate blood pressure in 87 study participants. The cuffless smartphone software showed acceptable accuracy for SBP and DBP when compared with the reference cuff-based manual blood pressure measurement method, according to the AAMI/ESH/ISO protocol criteria. However, we understand that this protocol was only designed to validate cuff-based blood pressure measuring devices. Therefore, further validation studies with a specific validation protocol designed for cuffless blood pressure measuring devices are required to assess clinical accuracy.

The cuffless smartphone technology can be easily applied in everyday life, and it may improve the awareness and control of hypertension in the general population by enabling regular blood pressure monitoring, remote monitoring by health care providers, and programmed alerts and reminders for medication and measurement. Additionally, educational content can promote adherence to treatment plans and provide tips for managing hypertension.

## Abbreviations

AAMI/ESH/ISO: Association for the Advancement of Medical Instrumentation/European Society of Hypertension/International Organization for Standardization
DBP: diastolic blood pressure
IMU: inertial measurement unit
MBP: mean blood pressure
PP: pulse pressure
PTT: pulse transit time
SBP: systolic blood pressure
